# Serology-Based Model for Personalized Epithelial Ovarian Cancer Risk Evaluation

**DOI:** 10.3390/curroncol29040220

**Published:** 2022-04-12

**Authors:** Tianqing Yan, Xiaolu Ma, Haoyun Hu, Zhiyun Gong, Hui Zheng, Suhong Xie, Lin Guo, Renquan Lu

**Affiliations:** 1 Department of Clinical Laboratory, Fudan University Shanghai Cancer Center, No. 270, Dong’An Road, Shanghai 200032, China; ytq140314@163.com (T.Y.); dadagis13@163.com (X.M.); beardyun@hotmail.com (H.H.); gongzhiyun2008@126.com (Z.G.); zh19841207xx@163.com (H.Z.); xiesuhong14@163.com (S.X.); guolin500@hotmail.com (L.G.); 2 Department of Oncology, Shanghai Medical College, Fudan University, Shanghai 200032, China

**Keywords:** epithelial ovarian cancer, serological indicators, risk model score, prognosis prediction

## Abstract

This study aimed to establish a prognosis-prediction model based on serological indicators in patients with epithelial ovarian cancer (EOC). Patients initially diagnosed as ovarian cancer and surgically treated in Fudan University Shanghai Cancer Center from 2014 to 2018 were consecutively enrolled. Serological indicators preoperatively were collected. A risk model score (RMS) was constructed based on the levels of serological indicators determined by receiver operating characteristic curves. We correlated this RMS with EOC patients’ overall survival (OS). Finally, 635 patients were identified. Pearson’s χ^2^ results showed that RMS was significantly related to clinical parameters. Kaplan–Meier analysis demonstrated that an RMS less than 3 correlated with a longer OS (*p* < 0.0001). Specifically, significant differences were perceived in the survival curves of different subgroups. Multivariate Cox analysis revealed that age (*p* = 0.015), FIGO stage (*p* = 0.006), ascites (*p* = 0.015) and RMS (*p* = 0.005) were independent risk factors for OS. Moreover, RMS combined with age, FIGO and ascites could better evaluate for patients’ prognosis in DCA analyses. Our novel RMS-guided classification preoperatively identified the prognostic subgroups of patients with EOC and showed higher accuracy than the conventional method, meaning that it could be a useful and economical tool for tailored monitoring and/or therapy.

## 1. Introduction

Ovarian cancer (OC) is the second most common cause of gynecological cancer-related death in women worldwide [1]. Epithelial ovarian cancer (EOC) is proposed as the most malignant gynecologic neoplasm, accounting for 90% of OC patients [2]. Additionally, two thirds of EOC patients are already in the advanced stage at the time of diagnosis [3]. Although most patients undergo primary surgery and platinum-based adjuvant chemotherapy, half of the patients will relapse within 16 months [4]. At present, blood-based prognostic biomarkers are rare, and the prognosis of EOC patients is mainly determined by the International Federation of Gynecology and Obstetrics (FIGO) stage and residual disease after cytoreductive surgery [5,6,7,8], which are limited to being confirmed after surgery or chemotherapy. Additionally, ovarian cancer constitutes a group of heterogeneous tumors based on distinctive morphological and molecular genetic features [2]. Thus, the individual prognosis of patients can be better assessed by integrating other significant prognostic factors. In recent years, great efforts have been made to identify new biomarkers through transcriptomics [9], genomics and epigenomics [10], proteomics [11], plasma exosomes [12] and lipidomics [13]. However, such detection panels are often time-consuming, uneconomical and difficult to apply in clinic applications. Therefore, the development of a widely available method becomes necessary for predicting the prognosis of ovarian masses, so as to guide the future therapeutic plans and surgical options.

In clinical practice, carbohydrate antigen 125 (CA125) and human epididymal protein 4 (HE4) are the most commonly used markers for the early diagnosis and prognostic prediction of EOC. However, the results are unsatisfactory [14,15,16]. Systemic inflammatory immunity markers such as the neutrophil-to-lymphocyte ratio (NLR), monocyte-to-lymphocyte ratio (MLR) and platelet-to-lymphocyte ratio (PLR) are economical and accessible for evaluation before initial treatment, and have been confirmed to play pivotal roles in the prognosis prediction of various cancers including EOC [17,18,19]. In addition, the fibrinogen-to-albumin ratio (FAR) and D-dimer were reported to be involved in tumor progression [20,21,22,23]. However, the research results of these indicators for predicting the prognosis of EOC are still limited. In addition, previous studies mainly focused on the effect of single serological indicator. Rare and conflicting studies evaluate the prognostic value through incorporating the expression pattern of multi-serological indicators.

Therefore, in the current study, we intend to establish a novel risk score model for prognosis prediction in EOC patients by integrating the tumor markers and systemic inflammatory immunity markers that are routinely detected preoperatively, which may be applied clinically to decision-making.

## 2. Patients and Methods

### 2.1. Patients

**Figure 1:**
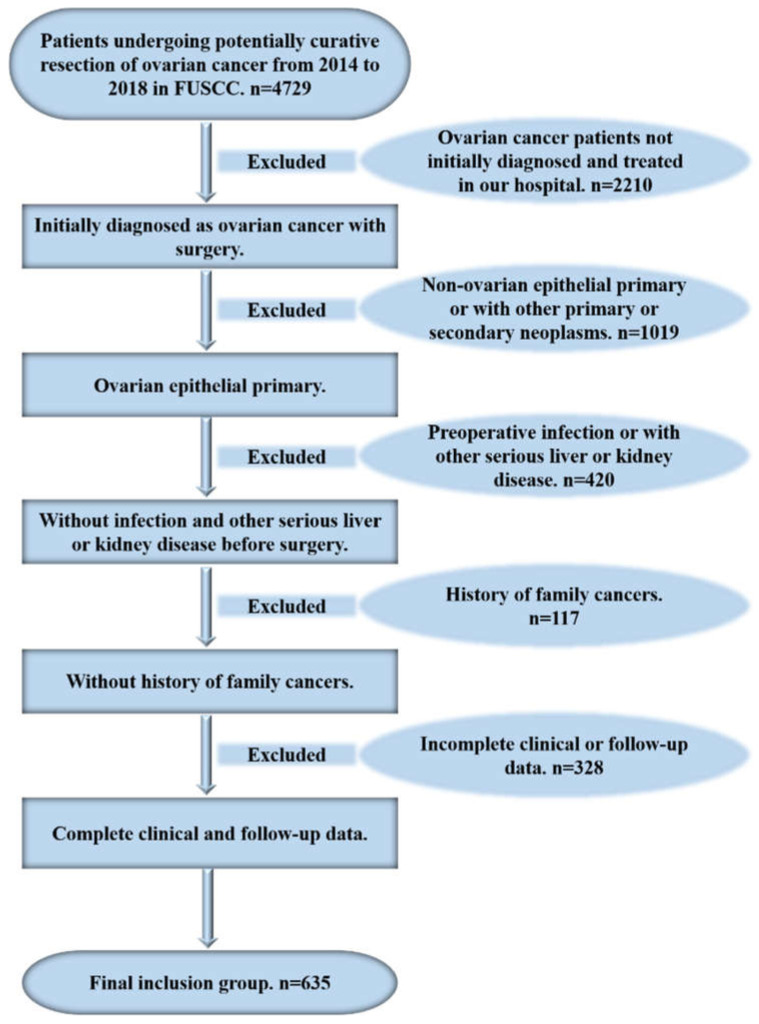
The flow chart of patients in the 5-year cohort to obtain the analytical sample.

In total, 635 female patients initially diagnosed pathologically as having EOC and surgically treated in Fudan University Shanghai Cancer Center (FUSCC) from 2014 to 2018 were consecutively enrolled in this retrospective observational study. All recruited patients achieved optimal debulking surgery, with the maximum diameter of residual tumor being less than 1 cm, followed by platinum-based chemotherapy. The inclusion criteria included patients initially diagnosed and pathologically confirmed as having OC in our center. The exclusion criteria included (1) patients not initially diagnosed and surgically treated in FUSCC; (2) non-ovarian epithelial primary or with other primary or secondary neoplasms; (3) preoperative infection or another serious liver or kidney disease; (4) a family history of tumors; and (5) incomplete clinical or follow-up data (Figure 1). This study was approved by the institutional research ethics committee of FUSCC (approval number 2019-Y022).

### 2.2. Data Collection

Data were collected retrospectively from our medical record database including age, body mass index (BMI), menopause status, FIGO stage, pathologic grades, histology, lymphatic metastasis, ascites volume and serological parameters, such as tumor markers, blood biochemical and coagulation indicators. Serum markers were measured within 1 week before ovariectomy. The automatic biochemical analyzer (Roche Cobas 8000/e 801/c 702), automatic blood cell analyzer (Sysmex XN9000) and automatic coagulation analyzer (Wolfen ACPTOP 750) were applied for detection. Follow-up was performed through a combination of active and passive patterns in three means. First of all, medical record follow-up (examination of the patients’ diagnoses and treatment information in FUSCC). Secondly, a phone visit within the time period specified in the follow-up plan. If the patient did not have any medical records, the patient or the pertinent family were contacted by the professional follow-up staff genus to ask and record relevant follow-up information. Thirdly, the date and cause of death were also obtained from the cause-of-death data link—a platform which links tumor registries to provincial centers for Disease Control and Prevention or the cause of death registration system. The final follow-up time of this study was 31 July 2021 and the median follow-up time (median, [interquartile range, IQR]) was 34.90 (24.83–50.53) months.

### 2.3. Definitions

Overall survival (OS), which is the primary outcome of this study, was calculated as the interval from the initial tumor resection to the date of death or censorship. NLR was defined as the absolute neutrophil count divided by the absolute lymphocyte count. MLR was defined as the absolute monocyte count divided by the absolute lymphocyte count. PLR was defined as the absolute platelet count divided by the absolute lymphocyte count. FAR was defined as the absolute fibrinogen count divided by the absolute albumin count. The optimal cut-off values for serological markers were assessed by receiver operating characteristic (ROC) curves. (Supplementary Table S2) and serological indicators lower than cut-off values were defined as “low expressed” and the value “0” was assigned, while those greater than cut-off values were defined as “high expressed” and the value “1” was assigned (Supplementary Figure S1). After that, a risk model score (RMS) was established according to the expression score of 7 serological markers (CA125, HE4, NLR, PLR, MLR, FAR, and D-dimer) as follows: a score of (1) 0 (non-high expressed among 7 serological markers); (2) 1 (single high expressed); (3) 2 (double high expressed); (4) 3 (triple high expressed); (5) 4 (four high expressed); (6) 5 (five high expressed); (7) 6 (six high expressed); and (8) 7 (all high expressed among 7 serological markers). Furthermore, patients were classified into two groups according to the score. The clinical characteristics and survival rates were analyzed based on the RMS. The other relative quantization parameters could be available in Supplementary Table S1.

### 2.4. Statistical Analysis

Statistical analyses were performed by SPSS (V.25.0, IBM, Armonk, New York, NY, USA) and R software (V.3.6.3. https://www.r-project.org/, accessed on 22 August 2021). The R packages “survminer”, “survival”, “rms”, “ggrisk” and “ggplot” with the appropriate libraries were used. The distribution of variables was assessed in total patient cohort. Continuous variables were described in the form of medians (IQR), and categorical variables were presented as numbers and proportions. Categorical variables were compared using the chi-squared test (Pearson’s χ^2^). Survival curves of different groups were estimated and compared by the Kaplan–Meier method and log-rank test. Univariate and multivariate Cox proportional hazards regression analyses were used to determine risk factors for prognosis of EOC and a nomogram model was applied. Then, internal validation was performed by simple bootstrapping, applying resampling with replacement 1000 times and exhibited by calibration curves. DCA curves were also developed to validate the clinical net benefit based on the Cox results.

## 3. Results

### 3.1. Demographic Parameters

In total, 635 patients were identified in this study and the demographic characteristics are shown in Table 1. The median age and BMI of the patients was 54 years (IQR: 48–62) and 23 kg/m^2^ (IQR: 20.70–24.50), respectively. A total of 410 (64.60%) patients were in the status of menopause. Based on the FIGO staging system, 497 (78.30%) patients were classified into stage III or IV, while 138 (21.70%) patients were grouped into stage I or II. A total of 544 (85.70%) patients belonged to the serous histology and 575 (90.60%) patients were in Grade 3. Overall, 175 (27.60%) patients were found to have tumor lymphatic metastasis and 208 (32.80%) patients had a volume of ascites more than 1000 mL. The serological indicators detected within one week before surgery were collected with a median serum CA-125 concentration of 521.00 U/mL (IQR: 161.50–1478.00), a median serum HE4 concentration of 285.80 pmol/L (IQR: 133.40–683.98), a median NLR of 2.90 (IQR: 2.00–4.15), a median PLR of 193.50 (IQR: 137.06–278.89), a median MLR of 0.27 (IQR: 0.20–0.38), a median FAR of 0.09 (IQR: 0.07–0.12), and a median serum D-dimer concentration of 3.16 μg/mL (IQR: 1.02–6.92). The median follow-up time of this study was 34.90 months (IQR: 24.83–50.53), and the median OS was 31.40 months (IQR: 21.30–47.63).

### 3.2. Clinical and Pathological Parameters Based on the RMS before Surgery

Patients were classified into two groups according to the RMS. Before the operation, 232 (36.5%) patients had scores of less than 3, while 403 (63.5%) patients had scores equal to or greater than 3. Table 2 shows that an RMS equal to or greater than 3 is significantly related to a more advanced FIGO stage (*p* < 0.0001), a higher pathologic grade (*p* < 0.0001), serous histology type (*p* < 0.0001), lymphatic metastasis (*p* < 0.0001) and larger amount of ascites (*p* < 0.0001).

### 3.3. Survival Rates Postoperatively Based on the RMS

**Figure 2:**
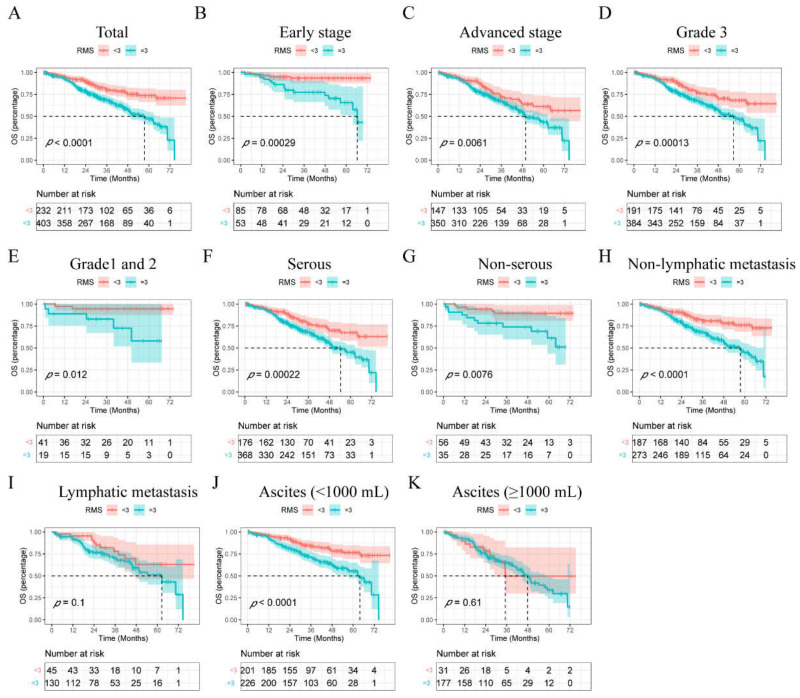
Kaplan–Meier analyses of OS in patients who underwent curative surgery for EOC. (**A**), Association of RMS preoperatively with OS in all patients who underwent curative surgery for EOC. (**B**,**C**) In patients with different FIGO stages. (**D**,**E**) In patients with different pathologic grades. (**F**,**G**) In patients with different histologic types. (**H**,**I**) In patients with different volumes of ascites. (**J**,**K**) In patients with different statuses of lymphatic metastasis.

Kaplan–Meier curves were employed to assess the effects of RMS on patients’ OS. The results show that the OS proportion decreased with an RMS equal to or larger than 3 before the operation (Figure 2A). Subgroup analyses showed significant differences in FIGO stage (early: *p* = 0.000, advanced: *p* = 0.006), grade (3: *p* = 0.000, 1 or 2: *p* = 0.012), histology (serous: *p* = 0.000, non-serous: *p* = 0.008), lymphatic metastasis (non-lymphatic metastasis: *p* < 0.0001) and ascites (volume < 1000 mL: *p* < 0.0001) based on RMS (Figure 2B–K).

### 3.4. Univariable and Multivariable Cox Regression Analyses of Parameters Related to OS in EOC Patients Undergoing Curative Resection

**Figure 3:**
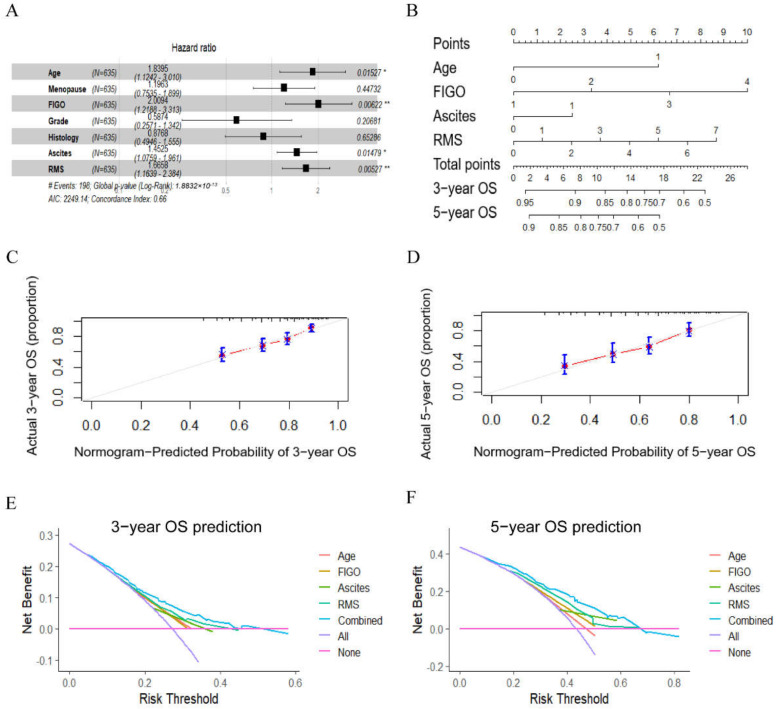
OS prediction based on RMS, age, FIGO stage and ascites volume. (**A**) Multivariate Cox analysis revealed age, FIGO, ascites and RMS to be independent risk factors for OS prediction. * *p* < 0.05, ** *p* < 0.01. (**B**) Nomogram to predict OS survival probability at 3 and 5 years. (**C**) Calibration curve for the nomogram predicting 3-year OS. (**D**) Calibration curve for the nomogram predicting 5-year OS. (**E**) DCA for the nomogram predicting 3-year OS. (**F**) DCA for the nomogram predicting 5-year OS. DCA, decision curve analysis.

RMS has been confirmed to be associated with the postoperative survival in EOC patients. Next, we examined whether RMS acted as an independent risk factor in EOC. Firstly, univariable Cox analysis proved that parameters including age, menopause, FIGO stage, pathologic grades, histology, ascites volume and RMS were risk factors of the patients’ OS (Supplementary Figure S3). Then, multivariable Cox analysis proved that age (HR = 1.840, *p* = 0.015), FIGO stage (HR = 2.009, *p* = 0.006), ascites (HR = 1.453, *p* = 0.015) and RMS (HR = 1.666, *p* = 0.005) before surgery were independent risk factors of the patients’ OS (Figure 3A). After that, a nomogram integrating age, FIGO, ascites and RMS was developed to visualize and test the efficiency of the model for the prognosis prediction of EOC patients (Figure 3B). The nomogram c-index was 0.677 (95% CI: 0.658–0.696), superior to that based on the FIGO stage alone (0.567, 95% CI: 0.553–0.581). The calibration curves also showed good consistency between the predicted and observed 3 and 5-year OS probabilities (Figure 3C,D). In addition, the clinical value of the nomogram model was evaluated using DCA, which provided the net benefits for a range of threshold probabilities. DCA showed that the novel nomogram which integrating RMS with age, FIGO stage and ascites added more benefit for postoperative survival prediction in EOC patients. Specifically, RMS showed even better performance than FIGO stage for the 3- and 5-year OS prediction (Figure 3E,F).

## 4. Discussion

In this retrospective cohort study, we constructed a risk evaluation model score (RMS) based on serum indicators that are routinely detected in EOC patients before surgery, which had a good discriminatory ability for postoperative survival in patients with EOC. Specifically, RMS could successfully stratify patients with different OS and higher RMS scores were associated with impaired prognoses. Furthermore, RMS combined with parameters including age, FIGO stage and ascites volume presented better performance for the prediction of prognoses in EOC patients.

OC is one of the most lethal gynecological cancers globally and is usually diagnosed at an advanced stage [1]. Up to now, there are no well-established prognostic factors, excluding FIGO stage and extent of debulking. However, these are limited to being confirmed after surgery. Instead, serology-based indicators preoperatively have the potential to be useful prognostic markers that may identify subsets of cancer patients who may benefit from tailored monitoring and/or therapy [24,25]. Additionally, using serology to identify potential poor prognoses may be useful as tumor tissue is not always available, while serology is not only minimally invasive and easy to obtain, but also economical and well applied. Moreover, association of multiple biomarkers may improve the validity of a prognostic test [20,26,27].

As common indices of early diagnosis before surgery and routinely monitored markers of prognosis in cancer, the prognostic values of serum CA125 and HE4 for EOC remain under investigation. Daniela et al., reported that in the training cohort of 136 women with EOC, both preoperative plasma HE4 and CA125 showed a good performance for identifying women at high risk of death from EOC. However, an external validation showed that HE4 but not CA125 was significantly associated with death in patients with serous tumors [28]. The optimal cut-offs of CA125 and HE4 they adopted are 282 U/mL (AUC: 0.631, sensitivity: 0.76, specificity: 0.51) and 277 pmol/L (AUC: 0.642, sensitivity: 0.75, specificity: 0.49), respectively. In our research, the cut-off value of HE4 is 340 pmol/L (AUC: 0.611, sensitivity: 0.60, specificity: 0.61), while CA125 is 284 U/mL (AUC: 0.590, sensitivity: 0.74, specificity: 0.41), close the values presented in previous reports [28].

Despite of studies on tumor-related antigens, inflammation has been reported to assist in cancer initiation and progression for years [27], and the prognostic significance of systemic inflammatory immunity markers, such as NLR, PLR, MLR [29], fibrinogen [30], D-dimer and albumin [21] has been of paramount interest. However, the data are contradictory. For example, different cut-offs of NLR ranging from 0.89 to 5.03 were used in studies [31]. The cut-off value of NLR applied in this study is 2.98, consistent with one previous report [32]. In addition, low serum albumin levels as an indicator of malnutrition status in patients have been validated to weaken the anti-tumor defense, leading to poor prognoses [20]. A composite indicator associating fibrinogen with albumin predicts the prognosis of EOC very well [33]. Therefore, it is reasonable to utilize an indicator composed of several parameters representing the systemic status of patients in a multidimensional manner, in order to denote the prognosis of patients with EOC.

In this study, we assessed the prognostic value of the combination of serological markers including CA125, HE4, NLR, PLR, MLR, FAR and D-dimer before surgery in EOC. For the purposes of reducing bias toward overestimation of the prognostic value of serum indicators, ROC curve analyses were performed to determine the optimal cut-off point of pretreatment each index, and RMS was established based on the levels of the indicators (Supplementary Table S2 and Figure S1). All patients were grouped according to the RMS, and significant differences were observed in the FIGO stage (*p* < 0.0001), pathologic grades (*p* < 0.0001), histology (*p* < 0.0001), lymphatic metastasis (*p* < 0.0001) and ascites volume (*p* < 0.0001) between the different RMS groups (Table 2).

Although the EOC patients of stage I have better prognoses than those of stage III or IV, the risk assessment and treatments are almost the same. Notably, only 20% of stage I EOC cases relapse and die, meaning that intensive treatment and closer follow-up needs to be paid for this category of individuals [34]. Thus, we further explored whether RMS could help to discriminate prognoses from different subgroups in EOC patients, particularly those in the same stage. Kaplan–Meier curves revealed that elevated RMS scores were associated with impaired OS in both total EOC patients and the pertinent subgroups (Figure 2). Moreover, a multivariable Cox analysis confirmed that age (HR = 1.840, *p* = 0.015), FIGO stage (HR = 2.009, *p* = 0.006), ascites (HR = 1.453, *p* = 0.015) and RMS (HR = 1.666, *p* = 0.005) before surgery were independent risk factors for patients’ OS (Figure 3A). Then, a nomogram integrating RMS, age, FIGO stage and ascites was developed to visualize and was internally validated using bootstrapping (Figure 3B). It was shown to have excellent calibration with a bootstrap corrected concordance index of 0.677 (95% CI: 0.658–0.696), superior to that based on the FIGO stage alone (0.567, 95% CI: 0.553–0.581). The calibration curves also showed good consistency between the predicted and observed 3- and 5-year OS probabilities (Figure 3C,D). In addition, DCA showed that the novel prognostic nomogram added more benefit compared to any single indicator alone. Intriguingly, RMS showed even better performance than FIGO stage for the 3- and 5-year OS prediction, which means that RMS could help the clinicians to evaluate the patients’ body condition preoperatively and perform precision analysis of each individual (Figure 3E,F). Additionally, risk plots were further applied to assess the Cox analysis results. The risk scores of all patients obtained by Cox regression were arranged in ascending order, and then a scatter plot was constructed, with the patient serial number on the *x*-axis and the risk scores or survival time on the *y*-axis. It can be seen that patients in the high-risk group had a shorter survival time (downward trend of scattered dots) and a higher mortality rate (more red dots) than those in the low-risk group (Supplementary Figure S4). These findings suggest that RMS improves the predictions of OS over clinicopathological factors alone in EOC, which could facilitate trial stratification, patient–doctor communication about prognosis, and clinical decision-making.

Summarily, the retrospective nature and potential selection bias are limitations of this study. In addition, some information regarding the detailed extent of debulking, which is an essential prognostic factor, was not available and was stratified, limiting the data that could be analyzed, and larger external studies are needed. Despite these limitations, our study investigated in EOC a novel prognostic assessment risk model (RMS) based on combined analyses of serological indicators, which showed more discriminately classified prognosis prediction than the FIGO staging system. We found that RMS was an independent risk factor of the postoperative survival in patients with EOC and showed higher accuracy than the conventional method in identifying the prognostic subgroups of EOC patients. Furthermore, the association of RMS with age, FIGO stage and ascites could be better predictive of the prognostic impact on OS. Preoperative RMS could be easily integrated into routine clinical practice, as it is a convenient, simple, and economical prognostic factor for risk stratification.

## Figures and Tables

**Table 1:** Baseline patient characteristics (*n* = 635).

Variables	Number of Patients
Age (years, median, IQR)	54 (48.00–62.00)
BMI (kg/m^2^, median, IQR)	23 (20.70–24.50)
Menopause (%)	
No	225 (35.40)
Yes	410 (64.60)
FIGO stage (%)	
Early	138 (21.70)
Advanced	497 (78.30)
Grade (%)	
G3	575 (90.60)
G1/G2	60 (9.40)
Histology (%)	
Serous	544 (85.70)
Non-serous	91 (14.30)
Lymphatic metastasis (%)	
No	460 (72.40)
Yes	175 (27.60)
Ascites (mL, %)	
<1000	427 (67.20)
≥1000	208 (32.80)
CA125 (U/mL, median, IQR)	521.00 (161.50–1478.00)
HE4 (pmol/L, median, IQR)	285.80 (133.40–683.98)
NLR (median, IQR)	2.90 (2.00–4.15)
PLR (median, IQR)	193.50 (137.06–278.89)
MLR (median, IQR)	0.27 (0.20–0.38)
FAR (median, IQR)	0.09 (0.07–0.12)
D-dimer (μg/mL, median, IQR)	3.16 (1.02–6.92)
Follow-up time (months, median, IQR)	34.90 (24.83–50.53)
OS time (months, median, IQR)	31.40 (21.30–47.63)

Abbreviations: BMI—body mass index; FIGO—International Federation of Gynecology and Obstetrics; NLR—ratio of neutrophils to lymphocytes; PLR—ratio of platelets to lymphocytes; MLR—ratio of monocytes to lymphocytes; FAR—ratio of fibrinogen to albumin; OS—overall survival.

**Table 2:** Relationships between preoperative RMS and clinical characteristics in patients with EOC (*n* = 635).

Variables	Risk Model Score (RMS)		
	<3 (*n* = 232)	≥3 (*n* = 403)	*p* Value
Age (years)			
<50	80(34.5)	124(30.8)	
≥50	152(65.5)	279(69.2)	0.335
BMI (kg/m^2^)			
<23	117(50.4)	182(45.2)	
≥23	115(49.6)	221(54.8)	0.200
Menopause			
No	85(36.6)	140(34.7)	
Yes	147(63.4)	263(65.3)	0.630
FIGO stage			
Early	85(36.6)	53(13.2)	
Advanced	147(63.4)	350(86.8)	<0.0001
Grade			
G3	191(82.3)	384(95.3)	
G1/G2	41(17.7)	19(4.7)	<0.0001
Histology			
Serous	176(75.9)	368(91.3)	
Non-serous	56(24.1)	35(9.7)	<0.0001
Lymphatic metastasis			
No	187(80.6)	273(67.7)	
Yes	45(19.4)	130(32.3)	<0.0001
Ascites (mL)			
<1000	201(86.6)	226(56.1)	
≥1000	31(13.4)	177(43.9)	<0.0001

## Data Availability

Data from this study are available to researchers who obtain permission from the corresponding author.

## Supplementary Materials

The following supporting information can be downloaded at: https://www.mdpi.com/article/10.3390/curroncol29040220/s1, Figure S1: The process of RMS establishment; Figure S2: Expressions of serological indicators routinely detected in EOC patients before surgery in the survival and the died; Figure S3: Univariable Cox regression analyses of parameters related to OS in patients with EOC undergoing curative resection; Figure S4: Risk plot demonstrating the age, RMS combined with FIGO stage and ascites for OS prediction; Table S1: Clinical parameters quantization in patients with EOC; Table S2: The optimal cut-off value of serological indicators determined by ROC.

## References

[1] Siegel R.L., Miller K.D., Fuchs H.E., Jemal A. (2021). Cancer Statistics, 2021. CA Cancer J. Clin..

[2] Kurman R.J., Shih Ie M. (2010). The origin and pathogenesis of epithelial ovarian cancer: A proposed unifying theory. Am. J. Surg. Pathol..

[3] Nash Z., Menon U. (2020). Ovarian cancer screening: Current status and future directions. Best Pr. Res. Clin Obs. Gynaecol..

[4] Berek J.S., Kehoe S.T., Kumar L., Friedlander M. (2018). Cancer of the ovary, fallopian tube, and peritoneum. Int. J. Gynaecol. Obstet..

[5] Muraji M., Sudo T., Iwasaki S., Ueno S., Wakahashi S., Yamaguchi S., Fujiwara K., Nishimura R. (2013). Histopathology predicts clinical outcome in advanced epithelial ovarian cancer patients treated with neoadjuvant chemotherapy and debulking surgery. Gynecol. Oncol..

[6] Pectasides D., Fountzilas G., Aravantinos G., Bamias A., Kalofonos H.P., Skarlos D., Briasoulis E., Konstantara A., Economopoulos T., Dimopoulos M.A. (2007). Epithelial ovarian carcinoma in younger vs older women: Is age an independent prognostic factor? The Hellenic Oncology Cooperative Group experience. Int. J. Gynecol. Cancer.

[7] Chang S.J., Hodeib M., Chang J., Bristow R.E. (2013). Survival impact of complete cytoreduction to no gross residual disease for advanced-stage ovarian cancer: A meta-analysis. Gynecol. Oncol..

[8] Du Bois A., Reuss A., Pujade-Lauraine E., Harter P., Ray-Coquard I., Pfisterer J. (2009). Role of surgical outcome as prognostic factor in advanced epithelial ovarian cancer: A combined exploratory analysis of 3 prospectively randomized phase 3 multicenter trials: By the Arbeitsgemeinschaft Gynaekologische Onkologie Studiengruppe Ovarialkarzinom (AGO-OVAR) and the Groupe d’Investigateurs Nationaux Pour les Etudes des Cancers de l’Ovaire (GINECO). Cancer.

[9] Millstein J., Budden T., Goode E.L., Anglesio M.S., Talhouk A., Intermaggio M.P., Leong H.S., Chen S., Elatre W., Gilks B. (2020). Prognostic gene expression signature for high-grade serous ovarian cancer. Ann. Oncol. Off. J. Eur. Soc. Med. Oncol..

[10] Fang F., Cardenas H., Huang H., Jiang G., Perkins S.M., Zhang C., Keer H.N., Liu Y., Nephew K.P., Matei D. (2018). Genomic and Epigenomic Signatures in Ovarian Cancer Associated with Resensitization to Platinum Drugs. Cancer Res..

[11] Pan J., Hu Y., Sun S., Chen L., Schnaubelt M., Clark D., Ao M., Zhang Z., Chan D., Qian J. (2020). Glycoproteomics-based signatures for tumor subtyping and clinical outcome prediction of high-grade serous ovarian cancer. Nat. Commun..

[12] Zhang W., Ou X., Wu X. (2019). Proteomics profiling of plasma exosomes in epithelial ovarian cancer: A potential role in the coagulation cascade, diagnosis and prognosis. Int. J. Oncol..

[13] Salminen L., Braicu E.I., Lääperi M., Jylhä A., Oksa S., Hietanen S., Sehouli J., Kulbe H., Bois A.D., Mahner S. (2021). A Novel Two-Lipid Signature Is a Strong and Independent Prognostic Factor in Ovarian Cancer. Cancers.

[14] Zhang M., Cheng S., Jin Y., Zhao Y., Wang Y. (2021). Roles of CA125 in diagnosis, prediction, and oncogenesis of ovarian cancer. Biochim. Biophys. Acta Rev. Cancer.

[15] Gentry-Maharaj A., Burnell M., Dilley J., Ryan A., Karpinskyj C., Gunu R., Mallett S., Deeks J., Campbell S., Jacobs I. (2020). Serum HE4 and diagnosis of ovarian cancer in postmenopausal women with adnexal masses. Am. J. Obstet Gynecol..

[16] Braicu E.I., Fotopoulou C., Van Gorp T., Richter R., Chekerov R., Hall C., Butz H., Castillo-Tong D.C., Mahner S., Zeillinger R. (2013). Preoperative HE4 expression in plasma predicts surgical outcome in primary ovarian cancer patients: Results from the OVCAD study. Gynecol. Oncol..

[17] Nakamoto S., Ikeda M., Kubo S., Yamamoto M., Yamashita T., Notsu A. (2021). Systemic immunity markers associated with lymphocytes predict the survival benefit from paclitaxel plus bevacizumab in HER2 negative advanced breast cancer. Sci. Rep..

[18] Templeton A.J., McNamara M.G., Šeruga B., Vera-Badillo F.E., Aneja P., Ocaña A., Leibowitz-Amit R., Sonpavde G., Knox J.J., Tran B. (2014). Prognostic role of neutrophil-to-lymphocyte ratio in solid tumors: A systematic review and meta-analysis. J. Natl. Cancer Inst..

[19] Feng Z., Wen H., Bi R., Ju X., Chen X., Yang W., Wu X. (2016). Preoperative Neutrophil-to-Lymphocyte Ratio as a Predictive and Prognostic Factor for High-Grade Serous Ovarian Cancer. PLoS ONE.

[20] Guo Y., Jiang T., Ouyang L., Li X., He W., Zhang Z., Shen H., You Z., Yang G., Lai H. (2021). A novel diagnostic nomogram based on serological and ultrasound findings for preoperative prediction of malignancy in patients with ovarian masses. Gynecol. Oncol..

[21] Chen W., Zhong S., Shan B., Zhou S., Wu X., Yang H., Ye S. (2020). Serum D-dimer, albumin and systemic inflammatory response markers in ovarian clear cell carcinoma and their prognostic implications. J. Ovarian Res..

[22] Watanabe A., Araki K., Harimoto N., Kubo N., Igarashi T., Ishii N., Yamanaka T., Hagiwara K., Kuwano H., Shirabe K. (2018). D-dimer predicts postoperative recurrence and prognosis in patients with liver metastasis of colorectal cancer. Int. J. Clin. Oncol..

[23] Lin Y., Liu Z., Qiu Y., Zhang J., Wu H., Liang R., Chen G., Qin G., Li Y., Zou D. (2018). Clinical significance of plasma D-dimer and fibrinogen in digestive cancer: A systematic review and meta-analysis. Eur. J. Surg. Oncol. J. Eur. Soc. Surg. Oncol. Br. Assoc. Surg. Oncol..

[24] Liu Z., Ge H., Miao Z., Shao S., Shi H., Dong C. (2021). Dynamic Changes in the Systemic Inflammation Response Index Predict the Outcome of Resectable Gastric Cancer Patients. Front. Oncol..

[25] Hu W., Yu J., Huang Y., Hu F., Zhang X., Wang Y. (2018). Lymphocyte-Related Inflammation and Immune-Based Scores Predict Prognosis of Chordoma Patients After Radical Resection. Transl. Oncol..

[26] Xu M., Wu Q., Cai L., Sun X., Xie X., Sun P. (2021). Systemic Inflammatory Score predicts Overall Survival in patients with Cervical Cancer. J. Cancer.

[27] Michels N., van Aart C., Morisse J., Mullee A., Huybrechts I. (2021). Chronic inflammation towards cancer incidence: A systematic review and meta-analysis of epidemiological studies. Crit. Rev. Oncol./Hematol..

[28] Furrer D., Gregoire J., Turcotte S., Plante M., Bachvarov D., Trudel D., Tetu B., Douville P., Bairati I. (2019). Performance of preoperative plasma tumor markers HE4 and CA125 in predicting ovarian cancer mortality in women with epithelial ovarian cancer. PLoS ONE.

[29] Starzer A.M., Steindl A., Mair M.J., Deischinger C., Simonovska A., Widhalm G., Gatterbauer B., Dieckmann K., Heller G., Preusser M. (2021). Systemic inflammation scores correlate with survival prognosis in patients with newly diagnosed brain metastases. Br. J. Cancer.

[30] Polterauer S., Grimm C., Seebacher V., Concin N., Marth C., Tomovski C., Husslein H., Leipold H., Hefler-Frischmuth K., Tempfer C. (2009). Plasma fibrinogen levels and prognosis in patients with ovarian cancer: A multicenter study. Oncologist.

[31] Ethier J.L., Desautels D.N., Templeton A.J., Oza A., Amir E., Lheureux S. (2017). Is the neutrophil-to-lymphocyte ratio prognostic of survival outcomes in gynecologic cancers? A systematic review and meta-analysis. Gynecol. Oncol..

[32] Badora-Rybicka A., Nowara E., Starzyczny-Słota D. (2016). Neutrophil-to-lymphocyte ratio and platelet-to-lymphocyte ratio before chemotherapy as potential prognostic factors in patients with newly diagnosed epithelial ovarian cancer. ESMO Open.

[33] Yu W., Ye Z., Fang X., Jiang X., Jiang Y. (2019). Preoperative albumin-to-fibrinogen ratio predicts chemotherapy resistance and prognosis in patients with advanced epithelial ovarian cancer. J. Ovarian Res..

[34] Calura E., Paracchini L., Fruscio R., DiFeo A., Ravaggi A., Peronne J., Martini P., Sales G., Beltrame L., Bignotti E. (2016). A prognostic regulatory pathway in stage I epithelial ovarian cancer: New hints for the poor prognosis assessment. Ann. Oncol. Off. J. Eur. Soc. Med. Oncol..

