# Supplementary material for: Serology-Based Model for Personalized Epithelial Ovarian Cancer Risk Evaluation
Source: Curr Oncol. 2022 Apr 12;29(4):2695–705. doi: 10.3390/curroncol29040220 (PMC9029686; doi:10.3390/curroncol29040220)
Supplement: Supplementary file 1 [file curroncol-29-00220-s001.zip › curroncol-1592834-supplementary.pdf]

Table S1. Clinical parameters quantization in patients with EOC.

Parameters	Quantization value
Age (year)	<50=0， ≥50=1
BMI (kg/m^2)	<23=0， ≥23=1
Menopause	No=0， Yes=1
FIGO stage	I+II=0， III+IV=1
Grade	Low differentiation=0， Medium or high differentiation=1
Histology	Serous=0， Non-serous (Mucinous、Clear cell carcinoma、Endometrioid carcinoma)=1
Lymphatic metastasis	No=0， Yes=1
Ascites (mL)	<1000=0， ≥1000=1

Table S2. The optimal cut-off value of serological indicators determined by ROC.

Indicators	Cut-off value	Sensitivity	Specificity	AUC
CA125	284 U/mL	73.7	40.7	0.590
HE4	340 pmol/L	60.1	61.3	0.611
NLR	2.98	58.1	57.0	0.573
PLR	242.6	44.4	71.2	0.577
MLR	0.27	66.2	53.8	0.613
FAR	0.09	68.2	51.5	0.591
D-dimer	1.76 μg/mL	73.7	41.6	0.575

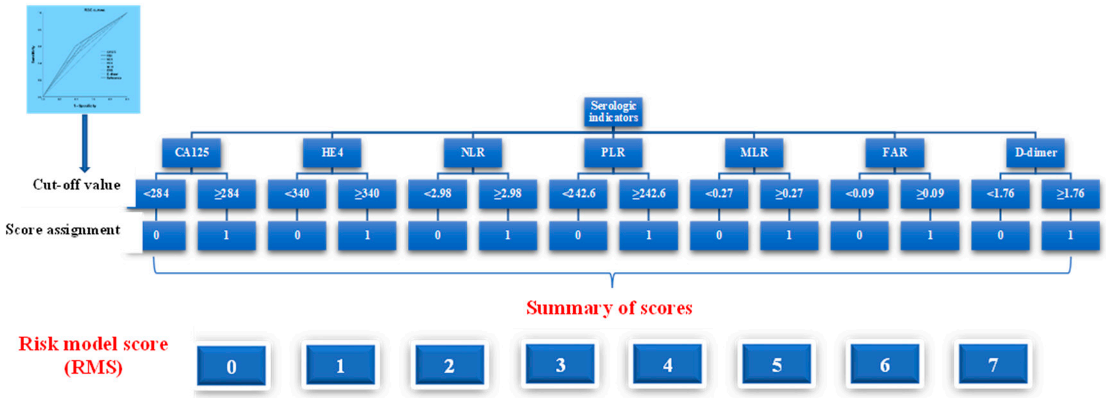

Figure. S1 The process of RMS establishment.

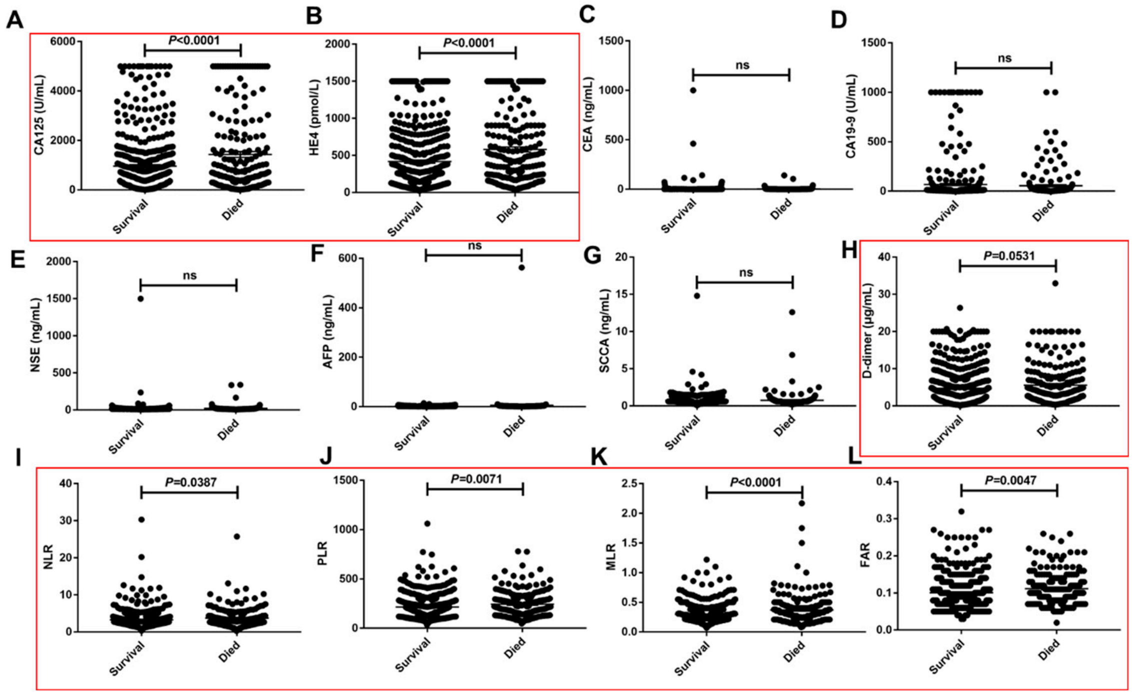

Figure. S2 Expressions of serological indicators routinely detected in EOC patients before surgery in the survival and the died. (Abbreviations: NLR, Neutrophile/lymphocyte, PLR, Platelet/lymphocyte, MLR, Monocyte/lymphocyte, FAR, Fibrinogen/albumin.)

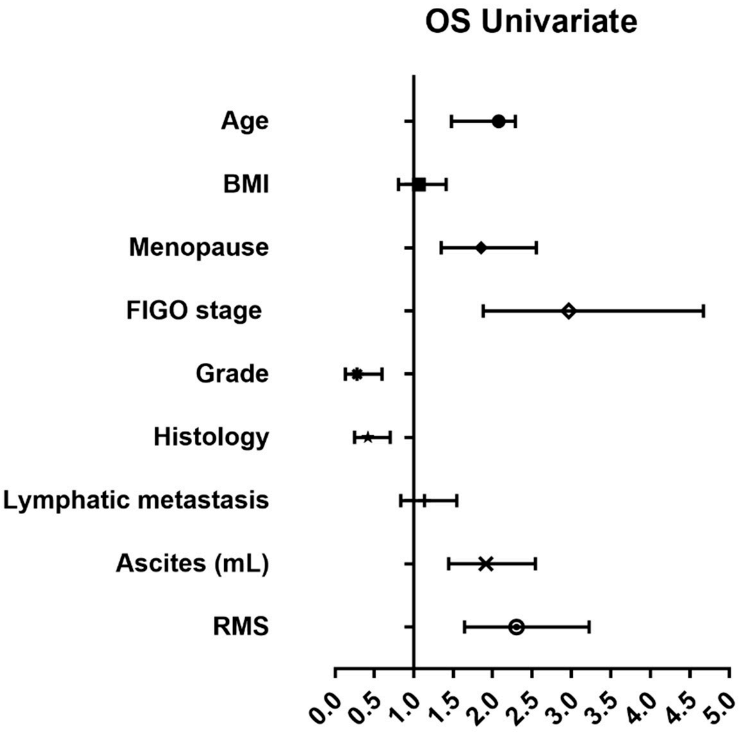

HR(95% CI)	*P* value
2.076(1.476-2.920)	<0.0001
1.065(0.805-1.409)	0.659
1.855(1.347-2.553)	0.000
2.967(1.884-4.672)	<0.0001
0.281(0.132-0.599)	0.001
0.418(0.250-0.699)	0.001
1.136(0.836-1.543)	0.416
1.916(1.443-2.543)	<0.0001
2.302(1.644-3.224)	<0.0001

Figure. S3 Univariable Cox regression analyses of parameters related to OS in patients with EOC undergoing curative resection.

Parameters with $P < 0.05$ in the univariable Cox regression analyses would be included in multivariable ones. Abbreviations: HR, hazard ratio; CI, confidence interval; OS, overall survival.

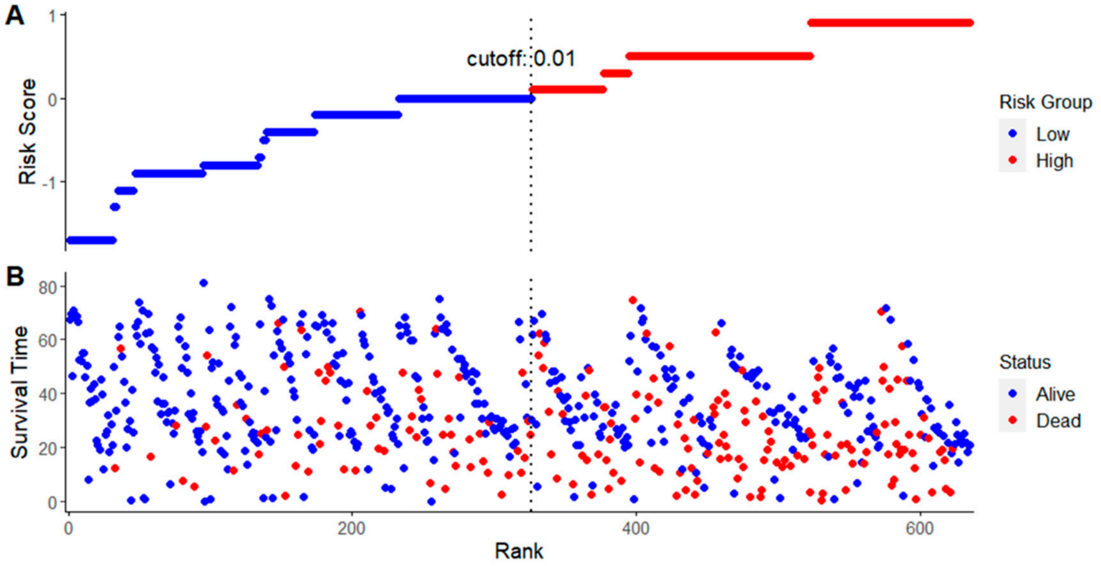

Figure. S4 Risk plot demonstrating the age, RMS combined with FIGO stage and ascites for OS prediction. A, Risk score distribution sorted by risk rank and classified by status. B, Survival time distribution sorted by risk rank and classified by status.
